# An observational study of hypertension treatment and patient outcomes in a primary care setting

**DOI:** 10.11604/pamj.2015.20.424.5040

**Published:** 2015-04-29

**Authors:** Keneuoe Hycianth Thinyane, Tumelo Mothebe, Mopa Sooro, Lekotoane David Namole, Varsay Cooper

**Affiliations:** 1 Department of Pharmacy, National University of Lesotho, Roma, Lesotho; 2 Queen Mamohato Memorial Hospital, Maseru, Lesotho

**Keywords:** Hypertension treatment, clinical outcomes, primary care

## Abstract

**Introduction:**

Evaluation and treatment of high blood pressure are vital to reducing hypertension-related morbidity. There are limited data on treatment of hypertension in Lesotho. The aim of this study was to investigate hypertension treatment and control in a primary care setting in Lesotho.

**Methods:**

A cross-sectional study was conducted among hypertensive patients treated at Domiciliary Health Clinic in Maseru, Lesotho between April and May 2013. We reviewed medical records and evaluated hypertension treatment and blood pressure control in the past 12 months. Patients were interviewed to assess adherence to hypertension treatment. Logistic regression analysis was used to identify factors associated with poor blood pressure control.

**Results:**

70 patients were enrolled in the study; 90.0% were female, the mean age was 57.7 years, 80.0% were overweight/obese and 27.1% had diabetes mellitus. 90.0% of the patients received combination antihypertensive therapy; the most frequently prescribed drugs were hydrochlorothiazide (90.0%), captopril (67.1%) and atenolol (51.4%). The majority of the patients had chronic uncontrolled hypertension. 67.2% of the patients had continuous access to antihypertensive drugs in the past 12 months; adherence to medication, diet and exercise was 64.3%, 37.1% and 7.1% respectively. Age ≥65 was the strongest independent predictor of poor blood pressure control (AOR = 10.3, 95% CI: 1.21-88.98, p = 0.033).

**Conclusion:**

There is a need for interventions to improve hypertension care and outcomes in this setting. Efforts should be made to improve assessment of hypertensive patients, optimise antihypertensive therapy and promote patient adherence to treatment.

## Introduction

Hypertension is a prevalent condition worldwide, contributing 4.5% of the global disease burden and 12.8% premature deaths annually [1, 2]. In a national survey conducted in Lesotho in 2001, the prevalence of hypertension was estimated to be 37.6% overall and 40.6% and 32.8% in women and men respectively. Consistent with existing literature, advanced age, overweight and obesity and physical inactivity were associated with a higher prevalence of hypertension [3]. Hypertension is a major cause of morbidity among adult patients in Lesotho; it is the third most common cause of outpatient attendance and one of the leading causes of admission to public health institutions [4]. Treatment of hypertension involves lifestyle changes and drug therapy with blood pressure lowering as the primary goal [5, 6]. Clinical practice guidelines for the management of hypertension recommend a stepwise approach for initiation of drug therapy. With this strategy, patients diagnosed with mild to moderate hypertension start antihypertensive drug treatment with monotherapy and treatment intensification is made when blood pressure targets are not met [5, 7]. Despite recent advances in drug therapy, worldwide the majority of diagnosed hypertensive patients are poorly controlled [8–10]. Reasons for inadequate control of hypertension are heterogeneous and include low adherence to anti-hypertension medication and lifestyle changes, low compliance with scheduled follow-up visits and suboptimal pharmacotherapy [11, 12]. In resource-poor settings, the cost of antihypertensive therapy is an important barrier to antihypertensive medication access and adherence [11, 13]. At present very little is known about hypertension treatment and blood pressure control among hypertensive patients in Lesotho. According to our unpublished observations, up to 90% of patients presenting to primary care clinics for hypertension follow-up treatment were poorly controlled. The aim of this study was to conduct a situation analysis of hypertension care and treatment outcomes in a primary care setting in Maseru, Lesotho.

## Methods

### Study design

We conducted a cross-sectional study among hypertensive patients treated at Domiciliary Health Clinic (DHC) over a 2 month period between April and May 2013.

### Setting

The study was conducted at Domiciliary Health Clinic, a primary health care clinic in Maseru, Lesotho. The clinic is one of more than 5 public primary health care centres in Greater Maseru, an area with an estimated population of 220,000. The DHC is located within a 2 km radius of the Maseru city centre and is accessible to patients from all areas of the city. In accordance with the national policy, patients pay a consultation charge of M15.00 (approximately $2) and all healthcare services and drugs are provided at no additional cost to the patient. During the study period, follow-up visits for hypertensive patients were scheduled on two days each week.

### Study population

The study population comprised adult patients who had been receiving antihypertensive treatment at Domiciliary Health Clinic for at least 12 months. Patients who were treated for hypertension exclusively at DHC over this time period were eligible for inclusion in the study. Patients were excluded if they had obtained treatment for hypertension elsewhere at any time in the past 12 months. Convenience sampling was used to select study participants. The purpose of the study was explained in Sesotho (the local language) to all patients who met the inclusion criteria and patients were then invited to participate in the study in consecutive order. All eligible patients who were willing to participate in the study were asked to give their written informed consent.

### Data collection

Data was collected on two days every week between April and May 2013 using a structured questionnaire designed specifically for this study. The questionnaire was developed with inputs from primary care physicians and pharmacists and pilot-tested among 20 hypertensive patients. Structured interviews were conducted with all study participants and the researchers filled out the questionnaires. Standard demographic and anthropometric data were obtained for each patient; standing height and weight were measured during the physical examination and were used to calculate the body mass index (BMI). We reviewed patients’ medical records for the past 12 months to obtain personal medical history on the diagnosis and treatment of hypertension and co-morbid conditions and documented the results of clinical and laboratory investigations performed within this time period. Patients were interviewed to determine their compliance with clinical visits, access to antihypertensive drugs and adherence to antihypertensive medication and lifestyle changes. Information provided by the patients on follow-up visits was cross-checked against the entries in their medical records.

### Data analysis

The data were captured and analysed using SPSS for Windows (Version 22.0). Internal validation was carried out to ensure data completeness. Data analysis was performed using descriptive statistics; results are summarised as count and percentages for qualitative variables and mean (±SD) for quantitative variables. Binary logistic regression was used to identify factors associated with poor blood pressure control; the outcome variable was uncontrolled blood pressure, covariates studied were age, body mass index, number of antihypertensive drugs used and adherence to medication. Adjusted odds ratios (AOR) and 95% confidence intervals (95% CI) were calculated; the level of significance was set at p < 0.05.

### Definitions

The following definitions were used in this study:

Patients were categorised as normal weight (BMI <25 kg/m^2^), overweight (BMI 25.0-29.9 kg/m^2^) or obese (BMI ≥30.0 kg/m^2^). Controlled hypertension was defined as a systolic BP of < 140 mmHg and a diastolic BP < 90 mmHg and uncontrolled hypertension as a systolic BP ≥140 mmHg and/or a diastolic BP ≥90 mmHg. Further classification of severity was undertaken according to the JNC7 [14] with stage I hypertension defined as BP ≥140/90 mmHg and stage II hypertension as BP ≥160/100 mmHg. A hypertensive crisis was defined as systolic BP ≥180 mmHg and/or diastolic BP ≥120 mmHg and resistant hypertension as a BP > 160/100 mmHg despite the use of at least 3 different antihypertensive drugs with complementary mechanisms of action, one of which being a diuretic. Access to antihypertensive drugs was evaluated for each patient in the past 12 months. Access was categorised as good if patients had continuous access to antihypertensive drugs and were able to obtain all of their prescribed drugs free of charge at the clinic during this period; fair if patients had continuous access to drugs but had incurred out-of-pocket expenses for antihypertensive drugs due to the unavailability of drugs at the clinic and poor if patients went without antihypertensive drugs and did not buy the unavailable drugs. Adherence to medicine was assessed using a 7 day self-report measure of adherence; good adherence was defined as taking all of the prescribed doses in the past 7 days and poor adherence as missing at least one dose for any reason. Study participants were considered to be adherent to exercise if they answered “Yes” to the question “Do you engage in at least 30 minutes of moderate intensity physical activity at least 4 times a week”? Adherence to diet was defined as responding “Yes” to the question “Do you follow a special diet for your hypertension”?.

### Ethics

Ethical approval for the study was obtained from the Lesotho Ministry of Health Research and Ethics Committee.

## Results

All patients who were invited to participate in the study gave consent. A total of 76 patients were enrolled; 6 patients were excluded due to missing medical records and 70 patients were included in the final analysis. 90.0% of the study participants were female and the mean age (± SD) was 57.7 (± 13.2) years, Table 1. The prevalence of overweight and obesity was 80.0%. 74.3% of the patients had been using antihypertensive drugs for 5 or more years and 61.4% had no compelling indications. The most common co-morbidity was diabetes mellitus, affecting 27.1% of the study participants. Less than one quarter of the study participants (21.4%) had controlled blood pressure; the proportions of patients presenting with stage I and stage II hypertension were 31.4% (n = 22) and 47.1% (n = 33) respectively. 9 patients (12.9%) presented with hypertensive crisis; the mean systolic/diastolic BP in the hypertensive crisis patients was 188/104 mmHg.

**Table 1:** Demographic and clinical characteristics^a^ of the study participants

Gender	Frequency,%
Female	90.0%
Male	10.0%
**Age groups, years**	
20 - 39	5.7%
40 - 55	38.6%
56 - 64	22.9%
≥ 65	32.9%
**BMI classification, kg/m** ^**2**^	
Normal weight	20.0%
Overweight	25.7%
Obese	54.3%
**Duration of hypertension**	
≥ 5 years	74.3%
< 5 years	25.7%
**Co-morbidities**	
Diabetes Mellitus	27.1%
Heart Failure	8.6%
**Evaluation of Blood pressure**	
Controlled blood pressure	21.4%
Uncontrolled blood pressure	78.6%
Hypertensive crisis	12.9%
Resistant hypertension	14.3%

a At the current clinical visit

Nearly all patients (69/70, 98.6%) were on diuretic-based antihypertensive regimens with 91.3% of these receiving hydrochlorothiazide, Table 2. Other frequently prescribed drugs included captopril (67.1%), atenolol (51.4%) and nifedipine (25.7%). 90.0% of the study participants were treated with a combination of two or more antihypertensive drugs. 32.9% of the patients had at least one change in antihypertensive drug regimen within the past 12 months: in 56.5% of these cases, antihypertensive drugs were switched because of side effects. The most frequently reported side effects leading to drug discontinuation were hypotension (n = 9, various drug combinations), fatigue in patients taking atenolol (n = 4) and captopril-induced cough (n = 2). Treatment intensification was carried out for 10 patients (43.5%) with elevated blood pressure. 67.2% of the patients had continuous access to antihypertensive drugs in the past 12 months; however only 52.9% of all study participants obtained all of the prescribed drugs free of charge from the clinic. Nearly one third of the study participants were categorised as having poor access to antihypertensive drugs; most reported that they could not afford to purchase drugs that were not available at the clinic as they were expensive.

**Table 2:** Hypertension treatment and patient self-care behaviours

Hypertension Treatment^a^	Frequency,%
***No. of drugs per regimen***	
1 drug	10.0%
2 drugs	37.1%
3 drugs	38.6%
≥ 4 drugs	14.3%
***Antihypertensive drug regimen***	
HCTZ	7.1%
HCTZ + captopril	17.1%
HCTZ + atenolol	12.9%
HCTZ + captopril + atenolol	24.3%
HCTZ + captopril + nifedipine	7.1%
HCTZ + captopril + atenolol + nifedipine	5.7%
Other combinations	25.7%
***Access to antihypertensive drugs***	
Good access	52.9
Fair access	14.3
Poor access	32.9
**Health care utilisation and self care behaviours**	
Total no. of clinic visits in the past 12 months	329
Average no. of visits/patient	4.7
***Attendance of scheduled follow-up visits***	
Regular	82.9%
Irregular	17.1%
***Patient self-management***	
Educated on hypertension self management	84.3%
Adherence to antihypertensive medications	64.3%
Adherence to diet	37.1%
Adherence to exercise	7.1%

a Antihypertensive drugs prescribed at the most recent clinical visit

Most patients had a 3-monthly hypertension follow-up schedule. The average number of clinical visits in the past 12 months was 4.7 visits/patient; the most common reasons cited for unscheduled clinic visits (n = 60) were development of other acute conditions and worsening of symptoms of hypertension and/or co-morbid conditions. The majority of the study participants had regular attendance of scheduled medical follow-up visits (delay of up to 7 days between the scheduled and actual clinic visit). 84.3% of the study participants had received education regarding hypertension self-management. However self-reported rates of adherence to medication, diet and exercise in the 7 days before the interview were low.

Table 3 is a summary of logistic regression analysis for variables predicting uncontrolled hypertension among the study participants. Factors associated with poor blood pressure control included advanced age and use of combination antihypertensive therapy. Age ≥65 years was the only statistically significant independent predictor of uncontrolled hypertension among the study population (AOR 10.3, 95% CI 1.21-88.98, p = 0.033). 68.4% of the patients with uncontrolled hypertension in this age group had combined systolic/diastolic hypertension.

**Table 3:** Factors associated with poor blood pressure control

Variable	AOR^a^	95% CI^b^
Age ≥ 65 years	10.3	1.21 - 88.98
***BMI***		
Normal		
Overweight	0.5	0.063 - 3.74
Obese	0.8	0.13 - 5.03
***No. of antihypertensive drugs/regimen***		
1 drug		
2 drugs	1.7	0.25 - 10.79
3 drugs	3.1	0.43 - 22.11
≥ 4 drugs	7.7	0.56 - 104.29
Non-adherence to medication	1.0	0.29 - 3.73

a AOR – adjusted odds ration

b 95% CI – 95% confidence interval

## Discussion

In this study conducted among 70 adult hypertensive patients attending follow-up treatment in a primary care setting in Lesotho, the study population was predominantly female, of advanced age (> 55 years) and overweight/obese. Less than a quarter of the study participants (21.4%) had adequately controlled blood pressure (<140/90 mmHg); the control rate for hypertension was even lower (15.7%) when the threshold level of 130/80 mmHg was applied to patients with diabetes mellitus and heart failure. These findings are consistent with data from other Sub Saharan Africa countries where control rates for hypertension ranging from as low as 2 to 40% for treated patients have been reported [9, 10, 13, 15]. Hypertension is a major risk factor for cardiovascular diseases (CVD) including ischemic heart disease, stroke and renal failure [6]. In this study, we found a high proportion of patients with chronic uncontrolled high blood pressure and other CVD risk factors including age > 55 (55.7%), obesity (47.1%) and diabetes mellitus (27.1%), however we were not able to determine the prevalence of dyslipidaemia as lipid profile tests were not performed routinely in all patients. These findings suggest a need to improve patient assessment and screening procedures to facilitate early detection and treatment of CVD risk factors and/or target-organ damage. Continuous care including uninterrupted access to and consistent use of antihypertensive drugs is integral to successful treatment of hypertension [5]. Although 67.2% of the patients in this study had continuous access to antihypertensive drugs in the previous 12 months, only 52.9% were able to obtain all of their prescribed drugs from the clinic; in cases of drug stock-outs at the clinic, 69.7% (23/33) of the affected patients did not purchase the unavailable drugs. Financial constraints are an important barrier to adequate management of chronic diseases including hypertension; patients with low incomes may be unable to make out-of-pocket payments for their healthcare including doctor visits and chronic medication [5, 11, 12]. Drug stockouts and shortages in public health facilities complicate hypertension treatment and may compromise patient outcomes [16]. Further studies are required to investigate incidences and causes of antihypertensive drug stockouts in this setting. In addition, several strategies including the referral of patients to other health centres can be put in place to minimise the impact of drug stockouts. Other factors contributing to treatment interruption among the study participants were non-adherence to medication (35.7%) and follow-up visits (17.1%). Hypertensive patients with higher medication adherence are more likely to attain blood pressure targets than non-adherent patients [5, 15, 17]. In this study, the majority of the patients had long term uncontrolled hypertension, however rates of treatment intensification were low. On the other hand, we found that patients receiving multiple drug antihypertensive therapy were more likely to have poor blood pressure control than patients on monotherapy. Furthermore, 14.3% of the patients were diagnosed with drug-resistant hypertension. Inadequate therapy including suboptimal drug dosing may account for inadequate treatment response and apparent resistance to therapy in treated hypertensive patients [18–20]. Implementing a systematic approach to the management of hypertensive patients can aid in identifying therapy-related problems including low medication adherence and inadequate pharmacotherapy. Further evaluation of patients, including the assessment of kidney function is also necessary to rule out secondary causes of hypertension among this population.

Lifestyle changes including dietary modification, increased physical activity and weight control lower blood pressure and may reduce the need for antihypertensive drugs, thus facilitating drug step down or withdrawal in patients with well-controlled hypertension [21, 22]. The use of fewer drugs or lower doses of drugs in combination therapy can minimise side effects associated with hypertension medication. Obesity, usually related to poor diet and low levels of physical activity, is common in hypertensive patients and BP control rates are lower in overweight and obese patients [9, 23–25]. In this study, 84.3% of the patients reported that they had received education on lifestyle modifications to control hypertension; however compliance with lifestyle changes was low. The adoption of healthy lifestyle behaviours in hypertensive patients is affected by the interplay between a number of demographic, psychosocial and cultural factors that hinder or encourage behavioural change [26]. Future research should aim to identify barriers to adopting lifestyle changes among this population and to develop strategies to overcome those barriers.

This study has several limitations. First, participation was limited to patients who had attended follow up hypertension treatment exclusively at Domiciliary Health Clinic. Evaluation of hypertension treatment and outcomes requires access to patients’ complete medical records over a specified period of time. While conducting the study, we found out that a significant proportion of patients had also sought hypertension treatment elsewhere in the previous 12 months. These patients were excluded due to lack of access to their medical data, thus reducing the number of eligible study participants. Secondly, the study setting was a primary healthcare clinic in an urban area of Maseru; the sociodemographic and clinical characteristics of hypertensive patients and the range of health care services offered may be different at other primary healthcare facilities in Lesotho thus limiting the generalisability of some of our findings. The main strengths of this study were the high response rate from eligible patients and access to detailed patient medical records which facilitated analysis of patient care and treatment outcomes over a period of several months. In addition, the use of concurrent patient interviews enabled us to study patient adherence to hypertension treatment and provided a preliminary insight into the issue of access to antihypertensive drugs among patients treated in a public primary care facility in Lesotho. Further research on the availability and affordability of antihypertensive medications in this setting and the impact this may have on treatment outcomes is warranted.

## Conclusion

In conclusion, the majority of the study participants were receiving diuretic-based combination antihypertensive drug therapy. There was a high prevalence of chronic uncontrolled high blood pressure; older age (≥ 65 years) and the use of multiple anti-hypertensive medications were independent predictors of poor blood pressure control. These findings suggest a need to evaluate the adequacy of antihypertensive drug therapy among this population and to implement treatment intensification when necessary. In addition patient assessment should be improved in order to facilitate the identification and management of hypertension -related complications and/or comorbid conditions which may complicate hypertension treatment. Future research should focus on developing patient directed interventions to improve adherence to medication and lifestyle changes.
